# Six-Month Outcomes in COVID-19 ICU Patients and Their Family Members: A Prospective Cohort Study

**DOI:** 10.3390/healthcare9070865

**Published:** 2021-07-08

**Authors:** Nadine van Veenendaal, Ingeborg C. van der Meulen, Marisa Onrust, Wolter Paans, Willem Dieperink, Peter H. J. van der Voort

**Affiliations:** 1 Department of Critical Care, University Medical Center Groningen, University of Groningen, P.O. Box 30.001, 9700 RB Groningen, The Netherlands; n.van.veenendaal@umcg.nl (N.v.V.); m.onrust@umcg.nl (M.O.); w.paans@pl.hanze.nl (W.P.); w.dieperink@umcg.nl (W.D.); p.h.j.van.der.voort@umcg.nl (P.H.J.v.d.V.); 2 Department of Anesthesiology, University Medical Center Groningen, University of Groningen, P.O. Box 30.001, 9700 RB Groningen, The Netherlands; 3 School of Nursing, Professorship Nursing Diagnosis, Hanze University of Applied Science, P.O. Box 3109, 9701 DC Groningen, The Netherlands; 4 TIAS School for Business and Society, Tilburg University, P.O. Box 90153, 5000 LE Tilburg, The Netherlands

**Keywords:** COVID-19, quality of life, post-ICU-syndrome, critical care, follow-up, PICS, PICS-F, physical functioning, psychological functioning, social functioning

## Abstract

Background: The COVID-19 pandemic has resulted in a major influx of intensive care unit (ICU) admissions. Currently, there is limited knowledge on the long-term outcomes of COVID-19 ICU-survivors and the impact on family members. This study aimed to gain an insight into the long-term physical, social and psychological functioning of COVID-19 ICU-survivors and their family members at three- and six-months following ICU discharge. Methods: A single-center, prospective cohort study was conducted among COVID-19 ICU-survivors and their family members. Participants received questionnaires at three and six months after ICU discharge. Physical functioning was evaluated using the MOS Short-Form General Health Survey, Clinical Frailty Scale and spirometry tests. Social functioning was determined using the McMaster Family Assessment Device and return to work. Psychological functioning was assessed using the Hospital Anxiety and Depression Scale. Results: Sixty COVID-19 ICU-survivors and 78 family members participated in this study. Physical functioning was impaired in ICU-survivors as reflected by a score of 33.3 (IQR 16.7–66.7) and 50 (IQR 16.7–83.3) out of 100 at 3- and 6-month follow-ups, respectively. Ninety percent of ICU-survivors reported persistent symptoms after 6 months. Social functioning was impaired since 90% of COVID-19 ICU-survivors had not reached their pre-ICU work level 6 months after ICU-discharge. Psychological functioning was unaffected in COVID-19 ICU-survivors. Family members experienced worse work status in 35% and 34% of cases, including a decrease in work rate among 18.3% and 7.4% of cases at 3- and 6-months post ICU-discharge, respectively. Psychologically, 63% of family members reported ongoing impaired well-being due to the COVID-19-related mandatory physical distance from their relatives. Conclusion: COVID-19 ICU-survivors suffer from a prolonged disease burden, which is prominent in physical and social functioning, work status and persisting symptoms among 90% of patients. Family members reported a reduction in return to work and impaired well-being. Further research is needed to extend the follow-up period and study the effects of standardized rehabilitation in COVID-19 patients and their family members.

## 1. Introduction

Since December 2019, coronavirus disease 2019 (COVID-19) has rapidly spread around the world, affecting more than 170 million people so far [1]. Like previous major outbreaks of viral infection in the 21th century, the COVID-19 pandemic is expected to have significant long-term clinical consequences for survivors [2,3,4].

While many infected individuals with COVID-19 suffer from mild illness with typical respiratory symptoms, a minority of patients are hospitalized [5]. Of these, 17–26% require admission to the intensive care unit (ICU) [6,7,8]. Severe respiratory failure and associated complications can have a major impact on the physical, cognitive and mental wellbeing of survivors after hospital discharge, known as the ‘post-intensive-care syndrome’ (PICS) [9,10]. Many ICU-survivors suffer from PICS, with 25–80% of survivors suffering from physical impairment, 8–57% from psychological problems, and 30–80% from cognitive impairment [11].

The impact of ICU-admission reaches beyond the patient. Family members of ICU-survivors can also suffer from symptoms, such as anxiety, depression, post-traumatic stress disorder (PTSD) and reduced quality of life (QoL). This phenomenon is known as post-intensive-care syndrome-family (PICS-F) [9]. As family members play an increasingly important role in the support of ICU patients, trust-building in the ICU setting is of paramount importance [12]. However, this was challenged during this pandemic due to the uncertainties of COVID-19, the limited opportunities for family members to visit their relatives in the ICU, and the new methods of digital contact.

Several studies have assessed the short-term outcomes of hospitalized COVID-19 patients [13,14,15,16,17,18,19], focusing mainly on patients who received ward-based care. Results show that age and comorbidities are associated with severe outcomes in COVID-19 patients [20,21]. However, there is limited data on the long-term effects of COVID-19. Moreover, the impact and consequences for family members are still unknown. In this study, we describe the physical, social and psychological impact on COVID-19 ICU-survivors and their family members at three- and six-months following ICU discharge.

## 2. Materials & Methods

The ‘COVID-19 Follow-up Intensive Care Studies’ (COFICS) aims to give insights into the long-term physical, social and psychological functioning of COVID-19 ICU-survivors and their family members. COFICS is a single-center, prospective cohort study, conducted at the ICU of the University Medical Center Groningen (Groningen, The Netherlands). Ethical approval for this study has been given by our hospital’s Medical Ethical Committee (METc 201800422), according to Dutch and European legislation. All patients gave their informed consent prior to data collection. The STROBE checklist for cohort studies was used to guide the study.

### 2.1. Study Participants

All COVID-19 patients admitted to the ICU between 19 March and 30 September 2020 and their family members were eligible to participate in this study. COVID-19 was diagnosed according to the World Health Organization (WHO) definition and was confirmed by RNA detection of SARS-CoV-2 using RT-PCR of oropharyngeal and nasopharyngeal swabs. Family members were partners, children, other close family members or friends who were identified by the patient as being important to them.

### 2.2. Procedure

All eligible patients were contacted by telephone by experienced research nurses for participation in this study, three months after discharge from the ICU. In addition, patients were asked if they agreed to have their family members contacted for participation. Multiple family members could participate per patient. Questionnaires were sent by ordinary mail at three and six months after ICU discharge. In case of no response, reminders were sent after three weeks. The results of lung-function tests were retrieved from local hospitals after obtaining informed consent from the patients for spirometry results.

### 2.3. Outcomes

Physical functioning was scored using the Dutch version of the 9-point Clinical Frailty Scale (CFS) [22]. The CFS consists of nine pictographs, ranging from ‘very fit’ (1) to ‘terminally ill.’ CFS-scores ranging from 1 till 4 were classified as ‘non-frail’ and from 5 to 9 as ‘frail’.

Physical functioning was additionally evaluated by requesting the results of spirometry tests at six-month follow-up. The following respiratory function parameters were measured: forced expiratory volume in 1 s (FEV1), forced vital capacity (FVC); forced expiratory ratio (FEV1/FVC), total lung capacity (TLC) and diffusing lung capacity for carbon monoxide (DLCO %). A measured value of more than 80% of the predicted value was considered normal [18].

Symptoms were collected by the question about which burdens were experienced by the patient at six months follow-up (Appendix A).

The MOS Short-Form General Health Survey (SF-20) was used to study general health outcomes related to physical and social functioning [23,24]. The SF-20 measures six QoL domains: physical functioning, role functioning, social functioning, mental health, general health perceptions and pain. The subscale mental health was not part of this study. The SF-20 total score was transformed linearly to a 0–100 scale where 0 represents the lowest and 100 the highest possible score.

Social functioning was evaluated with the general function scale of the McMaster Family Assessment Device (FAD-GF6+) [25]. The FAD-GF6+ is a short, validated version of the FAD, which is a quick and effective tool to assess the overall functioning of families [26]. The FAD-GF6+ identifies six dimensions of family life that are associated with a dysfunctional family. The total score is divided by the number of items on the subscale, giving a total score ranging from 1.0 (best functioning) to 4.0 (worse functioning) [27].

Return to work was measured as the proportion of previously employed ICU-survivors reporting return to work after critical illness, including work rate and change of work activities. Questions were derived from a systematic review on this topic [28]

Psychological functioning was measured using the Hospital Anxiety and Depression Scale (HADS) to study anxiety and depression [29]. The HADS contains a seven-item subscale for anxiety and a seven-item subscale for depression, with a four-point Likert scale for each question. Total scores per subscale ranged from 0 to 21, with the sums categorized as normal (0–7), mild (8–10), moderate (11–14) and severe (15–21). Fear of reinfection was questioned on a scale of 0 (‘no fear’) to 10 (‘high level of fear’). The influence of limited visiting possibilities was derived from a description of the family members regarding how they felt about the physical distances from their relative and if it had affected their well-being (Appendix A).

**Figure 1:**
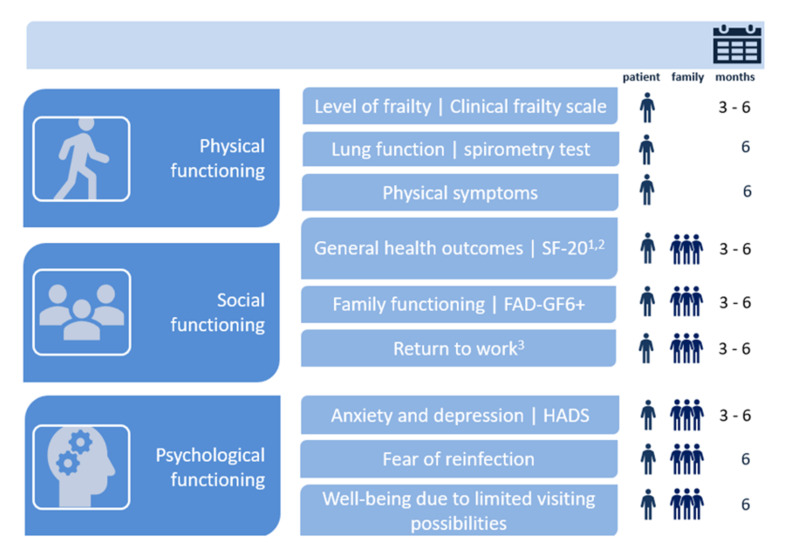
Overview of outcome variables per domain of the COFICS at three and six months post ICU discharge. ^1^ Subscale mental health is excluded. ^2^ Family members received these questions only at 3 months. ^3^ Patients received these questions only at 6 months.

An overview of the questionnaires used for this study is illustrated in Figure 1. Baseline patient characteristics, including age, gender and clinical data, such as length of hospital stay, comorbidities, delirium and age-adjusted Charlson Comorbidity Index (ACCI) [30], were retrieved from the patient’s electronic health record. Patient demographics, such as educational level and marital status, as well as healthcare consumption and family characteristics, were addressed in the three-month questionnaire.

### 2.4. Statistical Analysis

Quantitative data is reported as a median with an interquartile range (IQR), a mean with a corresponding standard deviation or a number with a percentage. Descriptive analyses were performed using IBM^®^ SPSS^®^ Statistics version 23.0 for Windows (New York, United States). The qualitative data was analyzed by two researchers (NV, IM) using ATLAS.ti version 9 for Windows (Berlin, Germany). The data was first coded inductively where the researcher used the words of the participant as a label (in vivo coding). Codes were categorized to a list of symptoms.

## 3. Results

### 3.1. Baseline Characteristics

**Figure 2:**
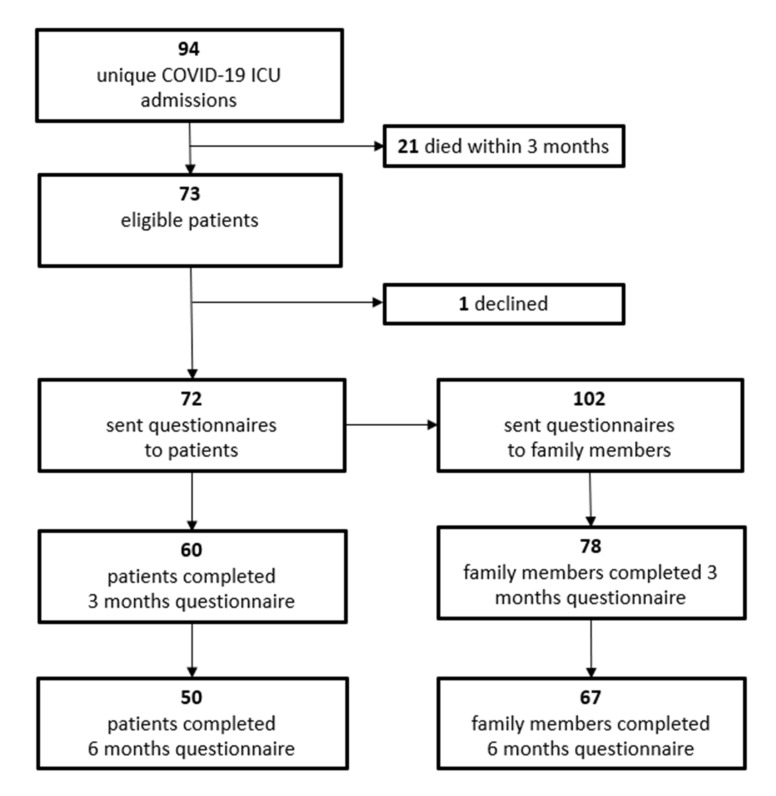
COFICS flow diagram: participant recruitment.

A total of 94 patients diagnosed with COVID-19 were admitted to the ICU during the study enrollment period. Seventy-three (78%) patients were alive and eligible for inclusion three months post ICU-discharge. Sixty (82%) COVID-19 ICU-survivors returned the 3-month questionnaire and 50 (68%) returned the 6-month questionnaire. A total of 102 family members of COVID-19 ICU-survivors were asked to participate in this study, of which 78 (76%) and 67 (66%) subjects completed the 3-month and 6-month questionnaire, respectively (Figure 2).

Participant and family member characteristics are given in Table 1A and Table 1B, respectively. Almost all ICU-survivors had a BMI above 25 (n = 56; 93%), and 55 (92%) ICU-survivors had an ACCI higher than 2. The median length of ICU stay was 19.4 days (IQR 12.3–31.7), of which 16.3 days (10.6–26.5) were on mechanical ventilation. Around half of the COVID-19 patients suffered from delirium during their ICU stay. Most of the family members were the partner of the patient, female or the median age was 56 years (IQR 41.0–63.0).

### 3.2. Results on Health Domains

#### 3.2.1. Physical Functioning

The physical functioning of COVID-19 ICU-survivors was poor three months post discharge, with a median score of 33.3 out of a maximum of 100 (IQR 16.7–66.7) on the physical functioning subscale and 35.0 (IQR 25.0–50.0) on experienced health. Scores slightly improved—but remained low—at six months with a median score of 50 (IQR 16.7–83.3) and 50.0 (IQR 35.0–71.3), respectively. Patients had a median pain score of 50 out of a maximum of 100 at three- and six-months follow-up. One-third of the ICU-survivors considered themselves ‘mildly frail’ to ‘frail’. At six months, FEV1, FVC and DLCO% were impaired in 18%, 20% and 69% of survivors, respectively (Table 2A). In addition, 90% of the ICU-survivors reported symptoms, such as fatigue, poor physical condition and polyneuropathy, at six months post discharge (Table 2A). ICU-survivors lost body weight, a median of 6.5 and 5.4 kg at three and six months, respectively, compared to pre-ICU admission.

Family members showed high levels of physical functioning with a median score of 100.0 (IQR 83.3–100.0) (Table 2B).

#### 3.2.2. Social Functioning

Role activities were impaired with a median of 0 (IQR 0–0) in ICU-survivors at three and six months. Social functioning scored a median of 60.0 (IQR 40.0–80.0) at three months and 80.0 (IQR 60.0–100.0) at six months. Family functioning showed high median scores of 4.0 (IQR 3.3–4.0) and 3.8 (IQR 3.2–4.0) at three and six months, respectively (Table 2A). Three (10%) of the 30 pre-ICU employed survivors fully returned to work, whereas 10 (43%) were still too ill to work at six months post ICU-discharge (Table 1 and Table 2A). Work rate was decreased for most patients at six months post ICU-discharge (Table 2A). Social functioning in family members scored high on role activities (median 100; IQR 50.0–100), social functioning (median 100; IQR 70.0–100) and family functioning (median 3.8; IQR 3.1–4.0). Of the 40 pre-ICU employed family members, 26 (65%) had fully returned to work at three months post ICU discharge, whereas nine (23%) were re-integrating, or had not yet returned to work. At six months, 23 of the 36 (64%) family members had fully returned to work, and 4 (11%) were re-integrating (Table 2B). Median work rate in family members decreased with 18.3% and 7.7% at three and six months, respectively, compared to pre-ICU admission. However, differences were observed between different family members (Table 1B and Table 2B).

#### 3.2.3. Psychological Functioning

Psychological functioning in ICU-survivors was good with median scores of ≤5.0 on the anxiety and depression subscales at three and six months (Table 2A).

Family members similarly showed good psychological functioning, with median scores of ≤4.0 on the anxiety and depression subscales at three- and six-months post-ICU (Table 2B). Fear of reinfection had a score of 5.0 for ICU-survivors and 6.0 for family members at six months.

Sixty-three percent of family members reported impaired well-being due to the mandatory physical distance from their relative at the time of ICU admission (Table 2B). Fifty-four (68%) family members were distressed by the physical distancing, and most described the situation as ‘very difficult’, ‘helpless’, ‘terrible’, ‘heavy’ and/or ‘terrifying’. In contrast, twelve family members were not distressed by the situation and mentioned that ‘the patient was in good hands’ or was ‘reasonably well through contact’. Most family members had contact with their loved ones via telephone and video during ICU-admission and described this as positive and supporting. In contrast, some family members experienced the telephone and video contact negatively, describing this as ‘tense’ or ‘difficult to get in contact’.

### 3.3. Health Care Consumption

All ICU-survivors received care by either a physiotherapist, general practitioner or pulmonologist during the follow-up period (Table 2A). Family members most commonly visited the general practicioner and social worker. (Table 2B).

## 4. Discussion

Our results show that COVID-19 ICU-survivors experienced limitations in physical functioning and reduced diffusion lung capacity, and 90% of patients reported at least one symptom after six months. Ninety percent of ICU-survivors had not reached their pre-ICU work level at six months post-discharge. In addition, 34% of family members experienced an impaired work status. Psychological functioning was normal in ICU-survivors and their family members. However, 63% of family members reported ongoing impaired well-being due to the COVID-19-related mandatory physical distancing from their relatives.

This study assessed the health consequences of both COVID-19 ICU-survivors and their family members at two follow-up times. It is known from previous coronavirus outbreaks, such as severe acute respiratory syndrome (SARS) and Middle East Respiratory Syndrome (MERS), that survivors suffer from pulmonary dysfunction, psychological impairment and reduced exercise capacity [2]. Survivors of acute respiratory distress syndrome (ARDS) are also known to experience a high prevalence of functional disability, cognitive impairment, post-traumatic stress disorder and impaired QoL [31,32,33,34]. Hence, high levels of physical, cognitive and psychosocial impairments among COVID-19 survivors are expected and require urgent attention.

Although most of the COVID-19 ICU-survivors in our study were impaired in the physical domain at 3- and 6-months follow-up, only a third of the patients considered themselves as ‘mildly frail’ to ‘frail’. A possible explanation might be the multidimensional concept of frailty, which includes the social and psychological domain as well [35].

DLCO was 80% lower than normal values in 69% of ICU-survivors. This percentage is higher than reported for general ward-based COVID-19 patients, where 22–47% had a reduced DLCO [18,36]. Our findings were in line with a subgroup with severe COVID-19 requiring invasive ventilation, in which 56% had an impaired DLCO [18]. It is likely that the gradual decrease in DLCO among COVID-19 survivors is associated with disease severity. Early lung function follow-up is warranted since respiratory rehabilitation can improve respiratory function [37].

The high cumulative incidence of symptoms, 90%, in our study cohort corresponds with previous studies that reported 74–87% symptoms in ward-patients [14,18] and 86% symptoms in ICU-patients [18]. New illness-related fatigue was the most common reported symptom, which corroborates other studies that had incidences of 53–81% [14,16,17,18]. Reduced physical condition was the second main symptom in our study, which overlapped with fatigue. Other studies reported breathlessness in 66% [17] and dyspnea in 42–43% [14,16] after COVID-19 infection. Yet, these symptoms were reported less frequently by the participants in our study.

Delayed return to work is common after critical illness and is likely a consequence of post-ICU impairments. After ICU-admission, 20–36% of survivor’s experienced job loss, 17–66% changed their occupation, and 5%-84% had a worsening of work status [29]. Pre-existing comorbidities are a potential risk factor for delayed return to work. Although 46% of the pre-ICU employed patients of our study cohort had returned to work at six months, many of these patients were not back to their pre-COVID-19 level, i.e., they worked fewer hours than before ICU admission. Our results correspond with the findings from previous studies in which 33–47% of ICU-survivors had returned to work at 3-months follow-up [16,17]. Therefore, COVID-19 recovery concerning return to work seems consistent with ICU-recovery due to other critical illness etiologies. Burden in the social domain for the family is reflected by on- third of the family members having not fully returned to work at 3 and 6 months. Despite limited evidence on this topic, it is known that 85% of ICU-patient caregivers had returned to their previous work level after one year [38,39].

Although psychological symptoms are likely to occur in two-thirds of acute respiratory distress syndrome survivors at 12-months follow-up [40], our results show no impairments in the psychological domain. A possible explanation for this can be a mixture of effective family support, reflected by the high mean family functioning and the relatively high health care consumption. The amount of family care compared to professional care was not studied, but this might be interesting for follow-up research.

In our study cohort, 100% and 96% of the patients consumed professional health care at 3 and 6 months follow-up, respectively. This is in line with 57% of patients needing healthcare assistance after prolonged mechanical ventilation at 1-year follow-up [41]. It is known that frail patients consume more health care services compared to patients who are not frail [42]. However, in our study cohort nearly all COVID-19 ICU-survivors consumed health care services at 6 months after ICU-discharge.

The responses of the family members in this study show that the profound nature of the situation was overwhelming. However, telephone and video contact were positively evaluated. Many family members (68%) declared that the physical distance was ‘very difficult’ to handle. However, it is known that the experiences of family members are not well-represented in existing standardized questionnaires [43].

The questionnaires used in COVID-19 studies are diverse, making comparisons between different studies difficult. An easy tool to evaluate the functional outcomes in COVID-19 survivors has been proposed but has yet to be validated [44]. In line, a standardized set of validated instruments in an international research context is recommended [45]. Standardized patient follow-up will benefit an organized way of determining functional recovery over time and can improve health care worldwide.

### Strengths and Limitations

A strength of this study is the relatively high response rate at follow-up, the inclusion of family members and the use of validated questionnaires and measurements. In addition, this study includes two follow-up time points after discharge, allowing comparisons over time.

We recognize that our study also has some limitations. First, the sample size of this prospective cohort study was limited, increasing the vulnerability to confounding factors. However, our sample size is larger than the ICU subgroups reported in previous studies [14,17,18]. Increasing the cohort size will allow stronger conclusions. Second, we did not assess the baseline physical and psychological status of the patients prior to development of COVID-19 infection neither did we have any information on pre-COVID-19 lung function (spirometry test outcomes). A proportion of the ICU-survivors had already experienced serious limitations to their physical, mental and cognitive functioning before ICU admission [46]. In addition, physical outcome was determined using questionnaires. Due to the COVID-19 restrictions during the study period, no objective measurements, for example the 6-min walking test, were possible. Furthermore, the cognitive domain was not tested in this study. Other studies showed the same limitations, as comprehensive studies including all three areas of PICS are lacking [47]. Finally, ICU-survivors were given an open question concerning symptoms instead of a questionnaire with predefined symptoms. This might have led to an underestimation of symptoms.

## 5. Conclusions

COVID-19 ICU-survivors suffer from a prolonged burden of disease, predominantly in physical and social functioning. At six months follow-up, 90% of ICU-survivors reported several symptoms, and 90% of ICU-survivors had not returned to their pre-COVID-19 work level. Among family members, 34% reported a worse work status and impaired wellbeing due to the physical distancing. Further research is needed to extend the follow-up and study effects of standardized rehabilitation on COVID-19 patients and their family members.

## Figures and Tables

**Table 1:** Characteristics of enrolled COVID-19 ICU-survivors and their family members.

(**A**)	
**Variable**	**ICU Survivors** **(n = 60)**
**Age**, years, median (IQR)	62.5 (55.3–68.0)
**Sex**, n (%)	
Male	41 (68)
Female	19 (32)
**Marital status**, n (%)	
Married/living together	51 (85)
Single	9 (15)
**Educational level ^1,2^**, n (%)	
Low	19 (32)
Middle	23 (38)
High	17 (28)
**Employment status ^3^**, n (%)	
Employed	30 (50)
Unemployed	20 (33)
**Work rate**, pre-admission (100% = full-time), mean (SD)	79.0 (28.7)
**Weight**, kg, median (IQR)	92.0 (83.0–104.3)
**BMI**, kg/m^2^, at ICU admission, median (IQR)	29.4 (26.6–32.4)
**BMI**, at ICU admission, n (%)	
Normal (18.5–25)	4 (7)
Overweight (25–30)	30 (50)
Obese (30–35)	19 (32)
Extremely obese (>35)	7 (12)
**APACHE IV**^2^, total score, median (IQR)	55.0 (45.0–65.3)
**Comorbidities**, n (%)	
Hypertension	20 (33)
Diabetes mellitus	15 (25)
Cardiovascular disease	12 (20)
Cerebrovascular disease	1 (2)
COPD/asthma	6 (10)
Chronic kidney disease	3 (5)
Malignancy	3 (5)
**ACCI**, n (%)	
0–1	5 (8)
2–3	28 (47)
≥4	27 (45)
**ECMO**, n (%)	
Yes	3 (5)
No	57 (95)
**Length of ECMO**, days, median (IQR)	21.7 (2.5)
**Mechanical ventilation**, days, median (IQR)	16.3 (10.6–26.5)
**Delirium during ICU-stay**, n (%)	
Yes	29 (48)
No	31 (52)
**Length of ICU stay**, days, median (IQR)	19.4 (12.3–31.7)
**Length of hospital stay**, days, median (IQR)	30.6 (21.9–44.7)
**Discharge location**^2^, n (%)	
Home	25 (42)
Other hospital	10 (17)
Nursing home	4 (7)
Rehabilitation center	20 (33)
(**B**)	
**Variable**	**Family** **(n = 78)**
**Relation to patient**, n (%)	
Partner	49 (63)
Son/daughter	22 (28)
Parent	5 (6)
Sibling	2 (3)
**Age**^2^, years, median (IQR)	56.0 (41.0–63.0)
**Sex ^2^**, n (%)	
Male	20 (26)
Female	57 (73)
**Marital status**, n (%)	
Married/living together	66 (85)
Single	12 (15)
**Educational level ^1^**, n (%)	
Low	16 (21)
Middle	38 (49)
High	24 (31)
**Employment status**, n (%)	
Employed	40 (51)
Unemployed	38 (49)
**Employment rate**, pre-admission (100% = full-time), mean (SD)	79.9 (26.5)

Abbreviations: IQR = interquartile range; SD = standard deviation; BMI = body mass index; APACHE IV = acute physiology and chronic health evaluation; ACCI = age-adjusted Charlson Comorbidity Index; ECMO = extracorporeal membrane oxygenation; ICU = intensive care unit ^1^ According to the ISCED2011 classification ^2^ Incomplete data ^3^ Return to work was only evaluated in the 6-months questionnaire; n = 50.

**Table 2:** The physical, social, and psychological functioning of **COVID-19 ICU survivors and their family members** 3- and 6-months post ICU-discharge.

(**A**)			
**Domain**	**Variable**	**3 Months** **(n = 60)**	**6 Months** **(n = 50)**
**Physical functioning**	Physical functioning ^1^ (range 0–100)	33.3 (16.7–66.7)	50.0 (16.7–83.3)
	Experienced health ^1^ (range 0–100)	35.0 (25.0–50.0)	50.0 (35.0–71.3)
	Pain ^2^ (range 0–100)	50.0 (25.0–75.0)	50.0 (0.0–75.0)
	Frailty, n (%)		
	Not frail (1–3)	40(67)	35 (70)
	Mildly frail (4–5)	17 (28)	14 (28)
	Frail (6–8)	3 (5)	0
	Lung function ^3^	-	
	FEV1 < 80%, % of predicted		7/39 (18)
	FVC L < 80%, % of predicted		7/35 (20)
	FEV1/FCV < 70%		1/36 (3)
	TLC < 80%, % of predicted		0
	DLCO < 80%, % of predicted		22/32 (69)
	Weight, kg	85.5 (80.3–93.5)	86.8 (80.5–95.0)
	Self-reported symptoms ^4^, n (%)	-	
	Any one of the following symptoms		44 (90)
	Fatigue		16 (33)
	Weakened condition		12 (25)
	Cognitive problems		9 (18)
	Polyneuropathy		7 (14)
	Impaired hand function		7 (14)
	Reduced lung function		5 (10)
	Dyspnea		5 (10)
	Difficulty walking		5 (10)
	Muscle weakness/stiffness		4 (8)
	Difficulty sleeping		3 (6)
	Shoulder pain		3 (6)
	Restriction of extremities		3 (6)
**Social functioning**	Role activities ^1^ (range 0–100)	0.0 (0.0–0.0)	0.0 (0.0–0.0)
	Social functioning ^1^ (range 0–100)	60.0 (40.0–80.0)	80.0 (60.0–100)
	Family functioning ^1^ (range 1–4)	4.0 (3.3–4.0)	3.8 (3.2–4.0)
	Return to work, n (%)	-	
	No change		3 (10)
	Reduced work rate		4 (13)
	Occupation change		3 (10)
	Re-integration		4 (13)
	Too ill to work		13 (43)
	Other		3 (10)
	Work rate (100% = full-time), mean (SD)	-	22.9 (32.2)
**Psychological functioning**	Anxiety (range 0–21)	3.5 (1.0–7.8)	4.5 (0.3–7.0)
	Depression (range 0–21)	4.0 (1.0–6.0)	2.0 (1.0–6.0)
	Fear of reinfection (range 1–10)	-	5.0 (2.0–7.5)
**Miscellaneous**	Health care consumption ^5^, n (%)		
	Yes	60 (100)	46 (92)
	No	0	4 (8)
	Readmission	5 (8)	5 (10)
	General practitioner	43 (72)	28 (56)
	Home care	12 (20)	3 (6)
	Physiotherapist	51 (85)	43 (86)
	Pulmonologist	42 (70)	32 (64)
	Psychologist	16 (27)	10 (20)
	Rehabilitation	25 (42)	0
	Dietician	8 (13)	1 (2)
(**B**)			
**Domain**	**Variable**	**3 Months** **(n = 78)**	**6 Months** **(n = 67)**
**Physical functioning**	Physical functioning ^1^ (range 0–100)	100 (83.3–100)	-
	Experienced health ^1^ (range 0–100)	70.0 (55.0–90.0)	-
	Pain ^2^ (range 0–100)	25.0 (0.0–50.0)	-
**Social functioning**	Role activities^1^ (range 0–100)	100 (50.0–100)	-
	Social functioning ^1^ (range 0–100)	100 (70.0–100)	-
	Family functioning ^1^ (range 1–4)	3.8 (3.1–4.0)	3.8 (3.2–4.0)
	Return to work ^6^, n (%)		
	No change	26 (65)	23 (64)
	Reduced work rate	1 (3)	5 (14)
	Occupation change	2 (5)	1 (3)
	Re-integration	5 (13)	4 (11)
	Not returned to work	4 (10)	0
	Job loss	1 (5)	0
	Unknown	1 (3)	3 (8)
	Work rate (100% = full-time), mean (sd)	61.6 (38.7)	72.5 (27.8)
**Psychological functioning**	Anxiety (range 0–21)	3.0 (2.0–7.0)	4.0 (1.0–8.0)
	Depression (range 0–21)	2.0 (1.0–6.0)	2.0 (1.0–6.0)
	Fear of reinfection (range 1–10)	-	6.0 (4.0–8.0)
	Influence no-contact on well-being, n (%)	-	
	Yes		42 (63)
	No		21 (31)
**Miscellaneous**	Health care consumption ^7^, n (%)		
	Yes	40 (51)	28 (42)
	No	38 (49)	39 (58)
	General practitioner	31 (40)	19 (28)
	Psychologist	9 (12)	8 (12)
	Social work	19 (24)	7 (10)

All numbers given are the median and interquartile range (IQR), unless otherwise stated. Abbreviation: ICU = intensive care unit ^1^ A higher score reflects a better functioning. ^2^ A higher score represents pain to a greater extent. ^3^ Data is given for 39 COVID-19 ICU-survivors (42 participants underwent a spirometry test, of which 1 participant did not consent to collect data from the hospital. For 2 participants, data was missing.) ^4^ n = 49 due to one non-responder. >5% reported symptoms are presented. Other burdens were mentioned (<5%), for example, balance problems, hair loss, smell/taste disorder, skin problems and/or hoarseness. ^5^ >5% reported health care consumption are presented. Other health care consumption was mentioned, for example, social work, occupational therapist, cardiologist, (vascular) surgeon or otorhinolaryngologists. ^6^ Employed family members at 3 months, n = 40, and at 6 months, n = 36. ^7^ Additionally, >5% reported health care consumption at 3 months are presented. Other health care consumption was mentioned, for example, home care, physiotherapist, company physician or coach.

**Figure A1:**
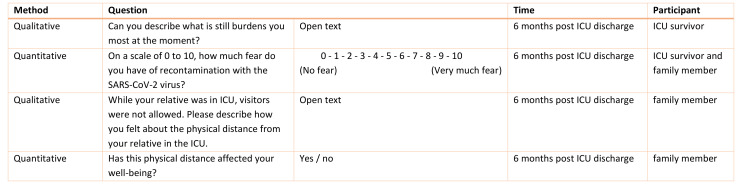
Overview of questions related to symptoms, fear of reinfection, post-influence of limited visiting possibilities and its influences on well-being.

## Data Availability

The datasets generated during and/or analyzed during the current study are available in the UMCG repository.

## Abbreviations

ACCI	Age-adjusted Charlson Comorbidity Index
ARDS	Acute respiratory distress syndrome
CFS	Clinical Frailty Scale
COVID-19	Coronavirus disease 2019
DLCO	Diffusing lung capacity for carbon monoxide
FAD-GF6	Family Assessment Device
FEV1	Forced expiratory volume in 1 s
FEV1/FVC	Forced expiratory ratio
COFICS	COVID-19 Follow-up Intensive Care Studies
FVC	Forced vital capacity
HADS	Hospital Anxiety and Depression Scale
ICU	Intensive Care Unit
MERS	Middle East Respiratory Syndrome
QoL	Quality of Life domains
SARS	Severe acute respiratory syndrome
SF-20	Short-Form General Health Survey
TLC	Total lung capacity
